# Effectiveness of different acupuncture courses for functional constipation: A protocol for systematic review and network meta-analysis

**DOI:** 10.1097/MD.0000000000020179

**Published:** 2020-05-22

**Authors:** Lu Wang, Dong Wang, Mingmin Xu, Wei Cao, Ying Liu, Tinghui Hou, Qianhua Zheng, Ying Li

**Affiliations:** a School of Acupuncture–Moxibustion and Tuina; b School of clinical medicine; c Graduate School, Chengdu University of Traditional Chinese Medicine, Chengdu, China.

**Keywords:** acupuncture, course, functional constipation, network meta-analysis, rotocol

## Abstract

**Background:**

This study will evaluate the effectiveness of different acupuncture courses for functional constipation (FC) through network meta-analysis.

**Methods:**

Eight database (PubMed, EMBASE, Web of Science, Cochrane Central Register of Controlled Trials (Central), China National Knowledge Infrastructure (CNKI), China Biomedical Literature Database (CBM) and Wanfang Database) will be searched from inception to October 2019. Only randomized controlled trials comparing different acupuncture courses or acupuncture versus sham acupuncture or placebo will be included. The outcomes involved weekly stool frequency, Bristol Fecal score, responder rate and safety evaluation. The risk of bias assessment and quality of evidence will be appraised using the Cochrane Risk of Bias Tool and the Grading of Recommendations, Assessment, Development and Evaluation guidelines. RevMan 5.3 software, STATA V.14.0 and GeMTC software will be used to perform the network meta-analysis.

**Results:**

This work will compare and arrange the comparative efficacy of different acupuncture treatments for FC by summarizing the current evidences. The results will be submitted in the form of a journal publication.

**Conclusion:**

The results of this network meta-analysis may help doctors determine the best treatments for patients to manage FC.

**PROSPERO registration number:**

CRD42020153801.

## 1 Introduction

According to the Rome IV criteria,^[1]^ functional constipation (FC) is characterized clinically by spontaneous defecation less than 3 times per week, incomplete defecation feeling, dry stool, and so on. In addition to digestive system symptoms or diseases, it can also cause anorectal disease, anorexia, diverticulosis, pain and mental symptoms.^[2–5]^ Although FC is not life-threatening, it makes a very significant adverse impact on the quality of life and economic costs of patients.^[6,7]^ Most epidemiological studies report prevalence in the general population between 12% to 19%,^[8–11]^ and female sex, reduced caloric intake, and increasing age are risk factors for FC.^[12,13]^ These negative impacts and high rates make FC a major public health issue.

The basic goal of FC treatment is to increase bowel frequency, alleviate the symptoms in patients with FC. Guidelines show management of constipation in stages. Commonly used to manage constipation manners include basic lifestyle and dietary modifications and pharmacological therapies. Increasing their fluid intake,^[14]^ supplementing the diet with fiber^[15]^ and exercising^[16]^ are recommended to improve bowel movement, but the strength of recommendation is weak.^[2]^ Medicines, such as osmotic and stimulant laxatives,^[17,18]^ secretagogues,^[19]^ serotonin(5-HT) 4 agonists^[20]^ and peripherally acting mu-opioid receptor antagonists,^[21]^ are reported with definite efficacy, but the majority of medicines have the adverse effects, such as drug-dependent, diarrhea, bloating, gas, bowel cramps, and esophageal obstruction.^[22]^ Therefore, the search for effective complementary and alternative therapies with few side effects for FC has attracted the attention of both doctors and patients.

Acupuncture is a treatment modality that originated in ancient China and has been used to treat various digestive diseases.^[23]^ Clinical and laboratory research have studied its application, along with electroacupuncture (EA), in constipation.^[24–26]^ Several studies have found that acupuncture may treat FC by regulating the nervous system and peripheral gastrointestinal hormones content.^[26–28]^ Moreover, some randomized controlled trials (RCTs) have proved that acupuncture can treat FC patients from multiple aspects, by increasing bowel movements, relieving symptoms, and improving quality of life.^[24,25,29–31]^ Whereas, we have found similar effects of different acupuncture courses in these RCTs.

However, there are no meta-analyses to compare 2 or more courses. Therefore, it is unclear what the optimal course of acupuncture is. Network meta-analysis can visualize large amounts of evidence, assess and arrange the comparative effectiveness of all intervention methods, despite lacking direct comparisons.^[32,33]^ Thus, this study will summarize the current evidences and conduct network meta-analysis to appraise the effectiveness of acupuncture in different courses for patients with FC.

## 2 Methods

The protocol will be performed on the basis of the PRISMA guidelines, the PRISMA network meta-analysis extension statement and the recommendations of the Cochrane Handbook.^[34–36]^ The registration number is CRD42020153801 in the PROSPERO.

### 2.1 Eligibility criteria

#### 2.1.1 Types of studies

The trials will include RCTs that used a 2-, or 3 -arm parallel design regardless of blinding. Other types of trials will be excluded, such as quasi-randomized trials, reviews, case reports, secondary analysis and animal researches.

#### 2.1.2 Types of participants

Patients over the age of 18 years who were diagnosed with FC using the Rome IV/III/II criteria will be included regardless of race, sex, education status or severity of disease. Patients can be included if they meet other clinical research guidelines and have no specific pathological cause. Studies with participants that included special populations, such as pregnant women, lactating women, addicts, strokes or those diagnosed with constipation due to irritable bowel syndrome, will be excluded.

#### 2.1.3 Types of interventions

##### 2.1.3.1 Acupuncture interventions

The experimental group should be treated by acupuncture or EA, without restrictions in terms of electrical stimulation intensity, acupuncture depth, the number of acupuncture points and treatment courses. However, the trials that the intervention group is acupuncture combines with other conventional therapy should be excluded.

##### 2.1.3.2 Comparison interventions

Through preliminary research, we have not found RCTs that directly compare different acupuncture courses. Therefore, we will indirectly compare the effects of different acupuncture courses. And sham acupuncture or placebo will be classified into a category as inert control. And trials simply comparing different acupuncture prescriptions or acupuncture forms will be excluded.

### 2.2 Outcomes of interest

#### 2.2.1 Primary outcomes

The primary outcome is weekly stool frequency (including spontaneous bowel movement (SBM), and complete SBM (CSBM)) at the end of all sessions. Specifically, a SBM refers to defecation without any medication or other means in the past 24 hours. A CSBM was defined as a SBM with the sense of complete evacuation.

#### 2.2.2 Secondary outcomes

The secondary outcomes involved stool consistency assessed with Bristol Fecal score, responder rate and safety evaluation. Bristol Fecal Scale uses 7 scales to evaluate stool characteristics, with scores of 1-2 indicating Lumpy or hard stools, 6 to 7 indicating mushy or entirely liquid stools. Responder rate is defined as the number of participants having at least 3 CSBMs or Bristol score of 3 to 5 per week. Safety evaluation is defined as the ratio of the number of reported adverse events to the total number.

### 2.3 Data sources and search strategy

We will systematically search electronic database from inception to October 2019, including 4 English databases: PubMed, Embase, Web of Science, Cochrane Central Register of Controlled Trials (Central), and 4 Chinese databases: China National Knowledge Infrastructure (CNKI), China Biomedical Literature Database (CBM) and Wanfang Database. According to patients, intervention and study type, the following search terms are used: constipation, FC, colonic Inertia, acupuncture, acupuncture therapy, acupuncture points, EA, randomized, randomized controlled trial and so on (Table 1 shows the search terms and strategy of PubMed). The other databases will use this search strategy with slight modification. And reference lists from other meta-analysis and all eligible papers will be reviewed for relevant studies.

**Table 1:**
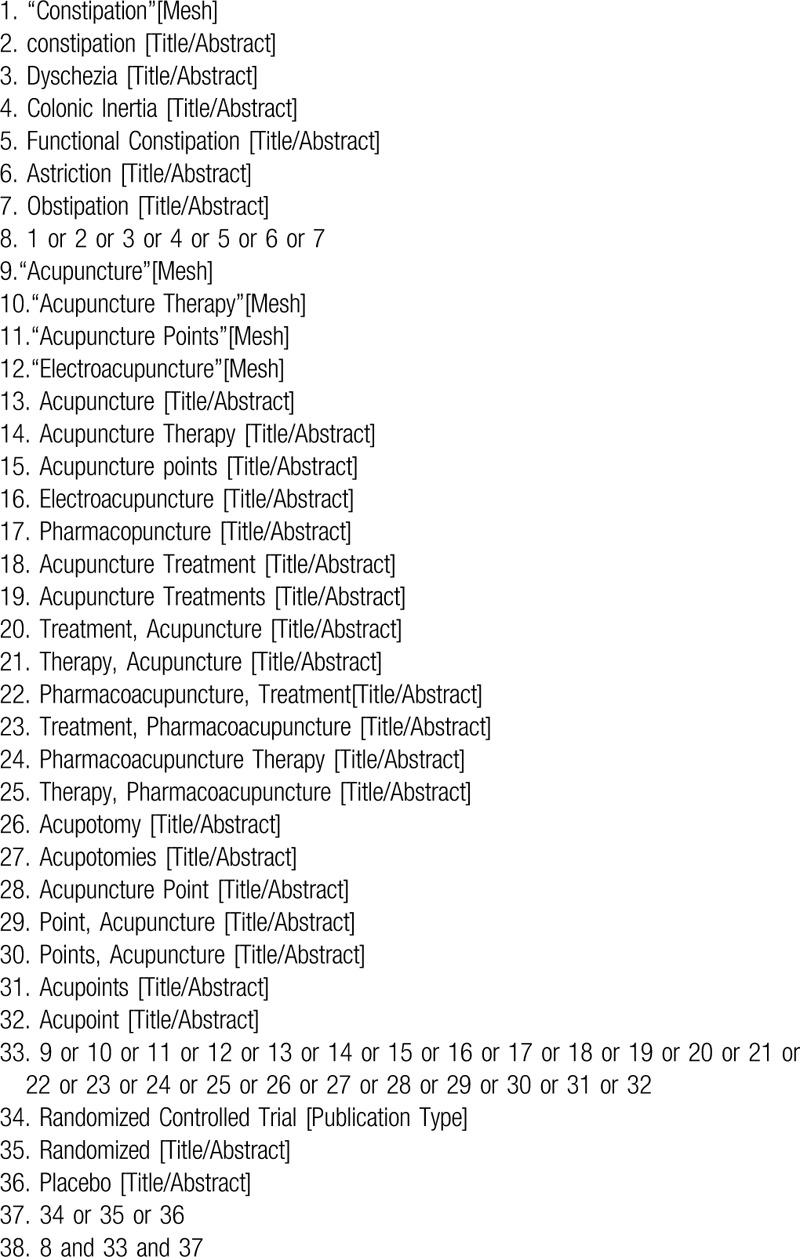
The search strategy in PubMed.

Two reviewers will conduct independent literature searches, and the objections will be negotiated or resolved by a third reviewer. Because of the language restriction of our researchers, only RCTs published in English and Chinese were included. The publication period and location of the study will not be limited.

### 2.4 Selection of studies and data extraction

**Figure 1:**
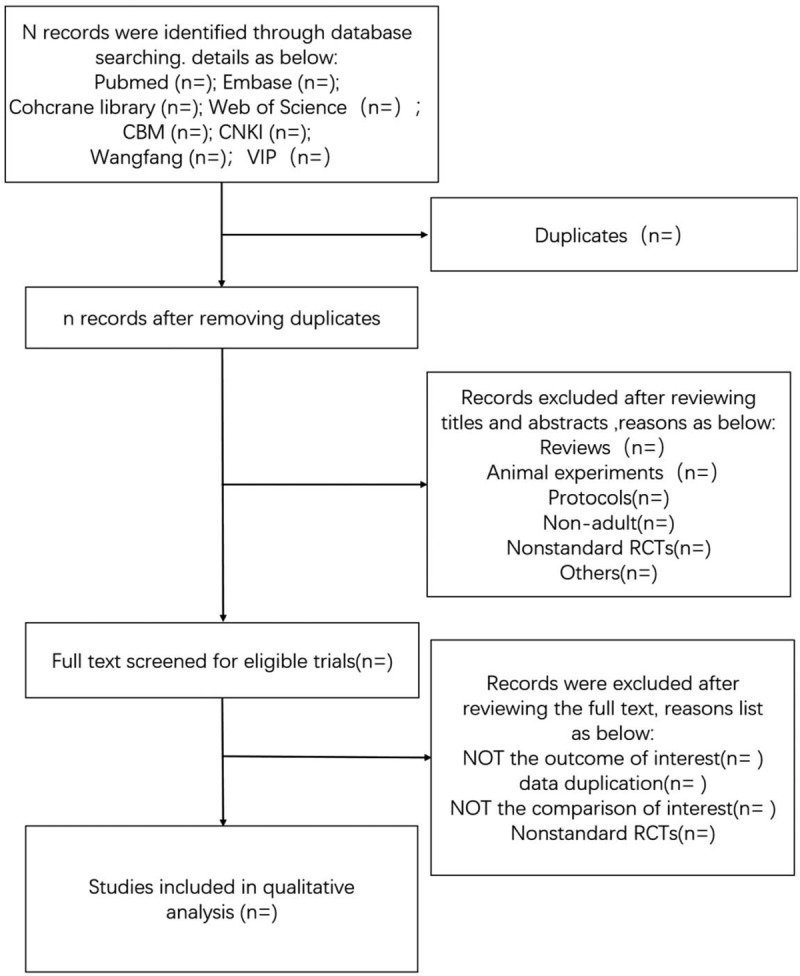
Flow diagram of the selection process.

The search results will be imported into endnote software for removing duplicate literature and managing. The preliminary study selection will be independently performed by 2 reviewers, who will screen the titles and abstracts of all included trials. And then reading the full text to determine if the article is included. Any disagreement will be discussed and decided by a third party. Then, Figure 1 shows the flow of this systematic review.

Two reviewers will independently examine eligibility for inclusion in the study and use Microsoft Excel to encode and extract data based on pre-designed data extraction forms after reaching a consensus. The Excel form includes general information (study sites, total numbers, numbers of acupuncture and control participants, acupuncture prescription, mean age, mean constipation duration, treatment duration, and outcomes). In the process, any inconsistencies will be discussed between the 2 reviewers and decided by a third party. Missing information was obtained by contacting the correspondent authors via e-mail.

### 2.5 Quality assessment

Two reviewers will evaluate the quality of included RCTs based on the Cochrane handbook V.5.1.0. The tool includes six domains: random sequence generation, allocation concealment, blinding of participants and personnel, blinding of outcome assessment, incomplete outcome data, selective reporting and other bias. They will be rated on 3 levels, “low risk”, “high risk” or “unclear”. Disagreements were resolved in consultation with the third reviewer. Moreover, we evaluated the quality of evidence for the outcomes of the included studies in our review using the Grading of Recommendations Assessment, Development and Evaluation guidelines, including 5 factors: study limitations, consistency of effects, indirectness, inaccuracy, and publication bias. Finally, the level of evidence for each outcome indicator can be divided into: high, moderate, low, and very low.^[37]^

### 2.6 Statistical analysis

First of all, we will use RevMan 5.3 software to perform the direct meta-analysis. For continuous variables, such as CSBM, SBM and Bristol score, the mean difference or standard mean difference with 95% confidence interval was used for analysis. For dichotomous data, such as the rates of responder s and adverse events, the relative risk (RR) with 95% confidence interval was utilized for analysis. If there are any data issues, we will deal with them according to the method described in the Cochrane handbook.^[38]^ The magnitude of heterogeneity was measured using the *I*^2^ statistic: when *I*^2^ < 50%, a fixed-effects model will be used for pooled data; and when *I*^2^≥50%, a random-effects model was used. A meta-analysis of indirect treatment comparisons will be retrieved from the available evidence if head-to-head comparisons are lacking.

Then, we will use STATA V.14.0 to make a network diagram and inconsistency test if there are 3 or more courses of treatment. The network diagram is showed by dots and lines. Among them, the dot represents a course of acupuncture with the size represents the number of subjects. The lines between the points indicate whether there is a direct comparison with the thickness represents the number of studies. And we will use the methods described in the previous article to test consistency.^[39]^

Finally, The indirect meta-analysis will be showed in the GeMTC software by using the Markov chain Monte Carlo method.^[40]^ We will use the Brooks-Gelman-Rubin method to assess convergence.^[41]^ Estimation and inference will be performed when the convergence state is stable. The GeMTC initial parameters will be set as follows: the number of chains is 4; the initial value is set to 2.5; the step size is 5; the number of simulation iterations is 100,000; the number of adjustment iteration is 20,000. Parameters can be adjusted appropriately according to specific conditions. With the potential scale reduced factor close to 1, we will determine whether the consistency of the model is reliable. Moreover, a ranking figure of all treatment sessions will be generated if multiple intervention times exist.

### 2.7 Subgroup or sensitivity analysis

Some factors may contribute to the heterogeneity, such as stimulation parameters, the severity of constipation, participants’ age or gender. When the *I*^2^>50%, subgroup or sensitivity analysis was performed to find the cause.

### 2.8 Assessment of reporting biases

When the number of included studies in each outcome is sufficient, we will use a funnel plot that evaluates the reported bias. (n>10)

## 3 Discussion

The factors affecting the efficacy of acupuncture in the treatment of FC are complex and diverse, of which the amount of acupuncture stimulation is the main factor, such as the selection of acupoints, acupuncture cycle, the depth of acupuncture, and the intensity of current.^[30,31,42]^ However, no systematic review on the comparison of different acupuncture courses has been published in the world. This systematic review will compare the curative effect of different acupuncture treatments on FC through network meta-analysis. We hope that this review will provide a reference for clinical decisions by acupuncturists.

## Author contributions

**Conceptualization:** Lu Wang.

**Data curation:** Mingmin Xu, Ying Liu, Tinghui Hou.

**Formal analysis:** Dong Wang, Wei Cao.

**Investigation:** Qianhua Zheng.

**Methodology:** Lu Wang, Mingmin Xu, Tinghui Hou.

**Project administration:** Lu Wang, Ying Liu.

**Supervision:** Ying Li.

**Writing – original draft:** Lu Wang, Wei Cao.

**Writing – review & editing:** Mingmin Xu, Ying Li.

## References

[1] Mearin F Lacy BE Chang L Bowel Disorders. Gastroenterology 2016;150:1393–407. 10.1053/j.gastro.2016.02.031 27144627

[2] Mearin F Ciriza C Minguez M Clinical practice guideline: irritable bowel syndrome with constipation and functional constipation in the adult. Rev Esp Enferm Dig 2016;108:332–63. 27230827 10.17235/reed.2016.4389/2016

[3] De Giorgio R Ruggeri E Stanghellini V Chronic constipation in the elderly: a primer for the gastroenterologist. BMC Gastroenterol 2015;15:130. 26467668 10.1186/s12876-015-0366-3 PMC4604730

[4] Andromanakos NP Pinis SI Kostakis AI Chronic severe constipation: current pathophysiological aspects, new diagnostic approaches, and therapeutic options. Eur J Gastroenterol Hepatol 2015;27:204–14. 25629565 10.1097/MEG.0000000000000288

[5] Sharma A Rao S Constipation: pathophysiology and current therapeutic approaches. Handb Exp Pharmacol 2017;239:59–74. 28185025 10.1007/164_2016_111

[6] Guerin A Carson RT Lewis B The economic burden of treatment failure amongst patients with irritable bowel syndrome with constipation or chronic constipation: a retrospective analysis of a Medicaid population. J Med Econ 2014;17:577–86. 24811855 10.3111/13696998.2014.919926

[7] Bruce Wirta S Hodgkins P Joseph A Economic burden associated with chronic constipation in Sweden: a retrospective cohort study. Clinicoecon Outcomes Res 2014;6:369–79. 25143749 10.2147/CEOR.S61985 PMC4136960

[8] Mugie SM Benninga MA Di Lorenzo C Epidemiology of constipation in children and adults: a systematic review. Best Pract Res Cl Ga 2011;25:3–18. 10.1016/j.bpg.2010.12.010 21382575

[9] Cheng C Chan AO Hui WM Coping strategies, illness perception, anxiety and depression of patients with idiopathic constipation: a population-based study. Aliment Pharmacol Ther 2003;18:319–26. 12895216 10.1046/j.1365-2036.2003.01663.x

[10] Suares NC Ford AC Prevalence of, and risk factors for, chronic idiopathic constipation in the community: systematic review and meta-analysis. Am J Gastroenterol 2011;106:1582–91. quiz 1581, 1592. 21606976 10.1038/ajg.2011.164

[11] Palsson OS Whitehead WE van Tilburg MA Rome IV diagnostic questionnaires and tables for investigators and clinicians. Gastroenterology 2016;150:1481–91. 10.1053/j.gastro.2016.02.014 27144634

[12] Chang JY Locke GR Schleck CD Risk factors for chronic constipation and a possible role of analgesics. Neurogastroenterol Motil 2007;19:905–11. 17988275 10.1111/j.1365-2982.2007.00974.x

[13] Dukas L Willett WC Giovannucci EL Association between physical activity, fiber intake, and other lifestyle variables and constipation in a study of women. Am J Gastroenterol 2003;98:1790–6. 12907334 10.1111/j.1572-0241.2003.07591.x

[14] Anti M Pignataro G Armuzzi A Water supplementation enhances the effect of high-fiber diet on stool frequency and laxative consumption in adult patients with functional constipation. Hepatogastroenterology 1998;45:727–32. 9684123

[15] Suares NC Ford AC Systematic review: the effects of fibre in the management of chronic idiopathic constipation. Aliment Pharm Ther 2011;33:895–901. 10.1111/j.1365-2036.2011.04602.x 21332763

[16] Gao R Tao Y Zhou C Exercise therapy in patients with constipation: a systematic review and meta-analysis of randomized controlled trials. Scand J Gastroenterol 2019;54:169–77. 30843436 10.1080/00365521.2019.1568544

[17] Ford AC Suares NC Effect of laxatives and pharmacological therapies in chronic idiopathic constipation: systematic review and meta-analysis. Gut 2011;60:209–18. 21205879 10.1136/gut.2010.227132

[18] Kamm MA Mueller-Lissner S Wald A Oral bisacodyl is effective and well-tolerated in patients with chronic constipation. Clin Gastroenterol Hepatol 2011;9:577–83. 21440672 10.1016/j.cgh.2011.03.026

[19] Li F Fu T Tong WD Lubiprostone is effective in the treatment of chronic idiopathic constipation and irritable bowel syndrome: a systematic review and meta-analysis of randomized controlled trials. Mayo Clin Pro 2016;91:456–68. 10.1016/j.mayocp.2016.01.015 27046523

[20] Camilleri M Piessevaux H Yiannakou Y Efficacy and safety of prucalopride in chronic constipation: an integrated analysis of six randomized, controlled clinical trials. Digest Dis Sci 2016;61:2357–72. 27056037 10.1007/s10620-016-4147-9 PMC4943977

[21] Luthra P Burr NE Brenner DM Efficacy of pharmacological therapies for the treatment of opioid-induced constipation: systematic review and network meta-analysis. Gut 2019;68:434–44. 29730600 10.1136/gutjnl-2018-316001

[22] Aziz I Whitehead WE Palsson OS An approach to the diagnosis and management of Rome IV functional disorders of chronic constipation. Expert Rev Gastroenterol Hepatol 2020;14:39–46. 31893959 10.1080/17474124.2020.1708718

[23] Schneider A Streitberger K Joos S Acupuncture treatment in gastrointestinal diseases: a systematic review. World J Gastroenterol 2007;13:3417–24. 17659687 10.3748/wjg.v13.i25.3417 PMC4146776

[24] Zhang T Chon TY Liu B Efficacy of acupuncture for chronic constipation: a systematic review. Am J Chin Med 2013;41:717–42. 23895148 10.1142/S0192415X13500493

[25] Liu ZS Yan SY Wu JN Acupuncture for chronic severe functional constipation a randomized trial. Ann Intern Med 2016;165:761–9. 27618593 10.7326/M15-3118

[26] Xiong F Wang Y Li SQ Clinical study of electro-acupuncture treatment with different intensities for functional constipation patients. J Huazhong Univ Sci Technolog Med Sci 2014;34:775–81. 25318892 10.1007/s11596-014-1351-8

[27] Liang C Wang KY Gong MR Electro-acupuncture at ST37 and ST25 induce different effects on colonic motility via the enteric nervous system by affecting excitatory and inhibitory neurons. Neurogastroent Motil 2018;30:e13318. 10.1111/nmo.13318 29488287

[28] Zhu XW Liu ZB Qu HY The effect and mechanism of electroacupuncture at LI11 and ST37 on constipation in a rat model. Acupunct Med 2016;34:194–200. 26561562 10.1136/acupmed-2015-010897 PMC4941155

[29] Da N Wang X Liu H The effectiveness of electroacupuncture for functional constipation: a randomized, controlled, clinical trial. Evid Based Complement Alternat Med 2015;2015:670963. 26064169 10.1155/2015/670963 PMC4433706

[30] Wu J Liu B Li N Effect and safety of deep needling and shallow needling for functional constipation: a multicenter, randomized controlled trial. Medicine 2014;93:e284. 25526462 10.1097/MD.0000000000000284 PMC4603109

[31] Wu X Zheng CH Xu XH Electroacupuncture for functional constipation: a multicenter, randomized, control trial. Evid Based Complement Alternat Med 2017;2017:1–0. 10.1155/2017/1428943 PMC5307003 28250788

[32] Gao Y Ge L Ma X Improvement needed in the network geometry and inconsistency of Cochrane network meta-analyses: a cross-sectional survey. J Clin Epidemiol 2019;113:214–27. 31150834 10.1016/j.jclinepi.2019.05.022

[33] Bafeta A Trinquart L Seror R Reporting of results from network meta-analyses: methodological systematic review. BMJ 2014;348:g1741. 24618053 10.1136/bmj.g1741 PMC3949412

[34] Moher D Liberati A Tetzlaff J Preferred reporting items for systematic reviews and meta-analyses: the PRISMA statement. BMJ 2009;339:b2535. 19622551 10.1136/bmj.b2535 PMC2714657

[35] Hutton B Salanti G Caldwell DM The PRISMA extension statement for reporting of systematic reviews incorporating network meta-analyses of health care interventions: checklist and explanations. Ann Intern Med 2015;162:777–84. 26030634 10.7326/M14-2385

[36] Higgins J Green S Cochrance Handbook for Systematic Reviews of Interventions Version 5.10[EB/OL]. The Cochrane Collaboration 2011. Available at: http://www.cochrane-handbook.org.

[37] Puhan MA Schunemann HJ Murad MH A GRADE working group approach for rating the quality of treatment effect estimates from network meta-analysis. BMJ 2014;349:g5630. 25252733 10.1136/bmj.g5630

[38] Cumpston M Li T Page MJ Updated guidance for trusted systematic reviews: a new edition of the Cochrane Handbook for systematic reviews of interventions. Cochrane Database Syst Rev 2019;10:ED000142. 31643080 10.1002/14651858.ED000142 PMC10284251

[39] Veroniki AA Vasiliadis HS Higgins JP Evaluation of inconsistency in networks of interventions. Int J Epidemiol 2013;42:332–45. 23508418 10.1093/ije/dys222 PMC5411010

[40] Caldwell DM Ades AE Higgins JP Simultaneous comparison of multiple treatments: combining direct and indirect evidence. BMJ 2005;331:897–900. 16223826 10.1136/bmj.331.7521.897 PMC1255806

[41] Brooks SP Gelman A General methods for monitoring convergence of iterative simulations. J Comput Graph Stat 1998;7:434–55.

[42] Zheng H Liu ZS Zhang W Acupuncture for patients with chronic functional constipation: a randomized controlled trial. Neurogastroenterol Motil 2018;30:e13307. 29392784 10.1111/nmo.13307

